# The impact of methicillin resistance on clinical outcome among patients with *Staphylococcus aureus* osteomyelitis: a retrospective cohort study of 482 cases

**DOI:** 10.1038/s41598-023-35111-w

**Published:** 2023-05-17

**Authors:** Hongri Wu, Chao Jia, Xiaohua Wang, Jie Shen, Jiulin Tan, Zhiyuan Wei, Shulin Wang, Dong Sun, Zhao Xie, Fei luo

**Affiliations:** Department of Orthopaedics, First Affiliated Hospital (Southwest Hospital), Army Medical University, Chongqing, 400038 The People’s Republic of China

**Keywords:** Clinical microbiology, Bacterial infection, Outcomes research

## Abstract

This study was designed to evaluate the impact of methicillin resistance on the outcomes among patients with *S. aureus* osteomyelitis. We reviewed all extremity osteomyelitis patients treated in our clinic center between 2013 and 2020. All adult patients with *S. aureus* pathogen infection were included. Clinical outcome in terms of infection control, length of hospital stay, and complications were observed at the end of a 24‐month follow‐up and retrospectively analyzed between populations with/without methicillin resistance. In total, 482 osteomyelitis patients due to *S. aureus* were enrolled. The proportion of methicillin-resistant *S. aureus* (MRSA) was 17% (82) and 83% (400) of patients had Methicillin-sensitive *S. aureus* (MSSA). Of 482 patients, 13.7% (66) presented with infection persistence after initial debridement and antibiotic treatment (6 weeks), needed repeated debridement, 8.5% (41) had recurrence after all treatment end and a period infection cure, complications were observed in 17 (3.5%) patients (pathologic fracture; 4, nonunion; 5, amputation; 8) at final follow-up. Following multivariate analysis, we found patients with *S. aureus* osteomyelitis due to MRSA are more likely to develop a persistent infection (OR: 2.26; 95% CI 1.24–4.13) compared to patients with MSSA. Patients infected with MRSA also suffered more complications (8.5% vs. 2.5%, p = 0.015) and longer hospital stays (median: 32 vs. 23 days, p < 0.001). No statistically significant differences were found in recurrence. The data indicated Methicillin resistance had adverse clinical implication for infection persistence among patients with *S. aureus* osteomyelitis. These results will help for patients counsel and preparation for treatment.

## Introduction

Extremity osteomyelitis is a notoriously refractory disease. The treatment typically consists of long courses of antibiotics in conjunction with surgical debridement of necrotic infected tissues^1–3^. Even with these standard measures, many patients go on to develop persistent infection. The challenge of its management is in part because of rising antibiotic resistance in causal pathogens. Among which, *S. aureus* is highly virulent and is the most common pathogen of extremity osteomyelitis^2,4,5^, rising methicillin-resistance is increasingly becoming a concern^1,6^. In comparison with *methicillin-sensitive S. aureus (*MSSA), *methicillin-resistance S. aureus* (MRSA) renders all β-lactams–type antibiotics inactive and these patients are typically more ill, which may complicate therapy.

Although MRSA is increasingly becoming an issue, the independent contribution of methicillin resistance to the outcomes for patients with *S. aureus* osteomyelitis is unclear. Currently, the majority of published studies have analyzed the impact of MRSA infection on clinical outcome (e.g. mortality, length of hospital stay) for bacteremia patients^7–9^. For extremity osteomyelitis patients, the comparison information between patients infected with MSSA and MSSA is limited. This is in part because of the small MRSA population in the series^10,11^. Only a few studies^12–14^ have indicated that osteomyelitis as a result of MRSA leads to an increased risk of relapse and complications. Moreover, most of these studies were simple descriptive studies that mainly focused on children. Patients infected with MRSA are typically older and sicker than patients infected with MSSA, which may confound clinical outcomes. To our knowledge, this is the first large cohort used to analyze attributable clinical outcome related to methicillin resistance among adult patients with *S. aureus* osteomyelitis.

The objective of this study was to evaluate the attributable impact of methicillin resistance on clinical outcomes among extremity osteomyelitis patients due to *S. aureus*. We hypothesized that there would be a difference in infection control and the lengths of hospital stay between patients infected with MRSA and those infected with MSSA. Our findings may advance the understanding and management of extremity osteomyelitis due to MRSA.

## Patients and methods

### Study design

This is a retrospective cohort study. From 2013 to 2020, we identified all patients with extremity osteomyelitis treated at our clinic center. This is a high-volume, level one bone infection treatment center, at a tertiary academic medical hospital. The treating team of specialists had abundant experience for osteomyelitis, with about 300 patients treated every year. The Ethics Committee of the First Affiliated Hospital of the Army Medical University approved this retrospective investigation, and all methods were performed in accordance with the relevant guidelines and regulations.

Inclusion criteria: extremity osteomyelitis; patients over 18; *S. aureus-*induced infection; and follow-up time ≥ 24 months. Exclusion criteria: not a curative or limb salvage treatment (palliation or amputation); incomplete medical record and follow-up data; patients with malignant disease. Then, patients were divided into two study groups; (1) osteomyelitis due to MRSA, (2) osteomyelitis due to MSSA.

### Definition and outcome measures

Clinical outcome in terms of infection control, lengths of hospital stay and related complication were followed up in the *S. aureus* osteomyelitis cohort (MRSA verse MSSA). The Infection control was evaluated by “infection persistence”^4,11^ and “recurrence”^15,16^ respectively. The infection persistence is defined as failure response to initial debridement and antibiotic treatment, that means patients had residual infection according to infection signs and symptoms, or imaging, pathological and serological tests, and needed repeated debridement. While osteomyelitis recurrence was defined as infection recurrent at the same site from which it had been previously eliminated, after all treatment end and a period of infection cure. The recurrence of osteomyelitis followed the consensus criteria on fracture-related infection (FRI)^15^ with one or more of: fistula, sinus or wound breakdown; purulent drainage from the wound or presence of pus during surgery; phenotypically indistinguishable pathogens identified by culture from at least two separate deep tissue/implant specimens; presence of microorganisms in deep tissue taken during an operative intervention, as confirmed by histopathological examination. Patients follow-up were conducted by one special fellowship-trained clinical researcher, through periodic telephone calls and outpatient follow-up visits.

The second outcome was lengths of hospital stay (total hospital days including readmissions). Treatment procedures and related complications were also recorded. Other demographic and clinical characteristics data were collected from medical electronic record used as potential confounders, including sex, age, infection site and duration, prior debridement, aetiology (hematogenous or secondary to trauma/fractures), tobacco use, Cierny-Mader host types, as well as systemic disease (e.g. diabetes, hypertension, chronic hepatic dysfunction, renal insufficiency, and immunosuppression). Moreover, CCI (charlson comorbidity index) score also was calculated as a potential influencing factor. Among which, microbiological and antimicrobial susceptibility results were obtained from the clinical microbiology database. *S. aureus* isolates were identified by growth of coagulase- and catalase-positive gram-positive cocci. Methicillin resistance was determined by lack of inhibition of growth by an oxacillin disc on mannitol salt agar, according to the criteria defined by the National Committee for Clinical Laboratory Standards.

### Statistical analysis

Differences in demographic factors, comorbidities, treatment procedures, infection control outcomes, lengths of hospital stay and complications between MRSA and MSSA populations were examined using the Chi-square test for category variables and the *t* test for continuous variables. The focal variable is the presence of methicillin resistance, a binary variable. Logistic regression analysis was used to determine the association between clinical outcome and each of the categorical variables. Variables with a significance probability of p < 0.25 were then included in the multivariate logistic regression analysis to further examine their effects. P values < 0.05 were deemed to be statistically significant using a two-sided test.

### Ethical approval

The Ethics Committee of the First Affiliated Hospital of the Army Medical University approved this retrospective investigation (KY201878).

### Consent to participate

Informed consent was obtained from all individual participants included in the study.

## Results

### Demographic and clinical data

**Figure1:**
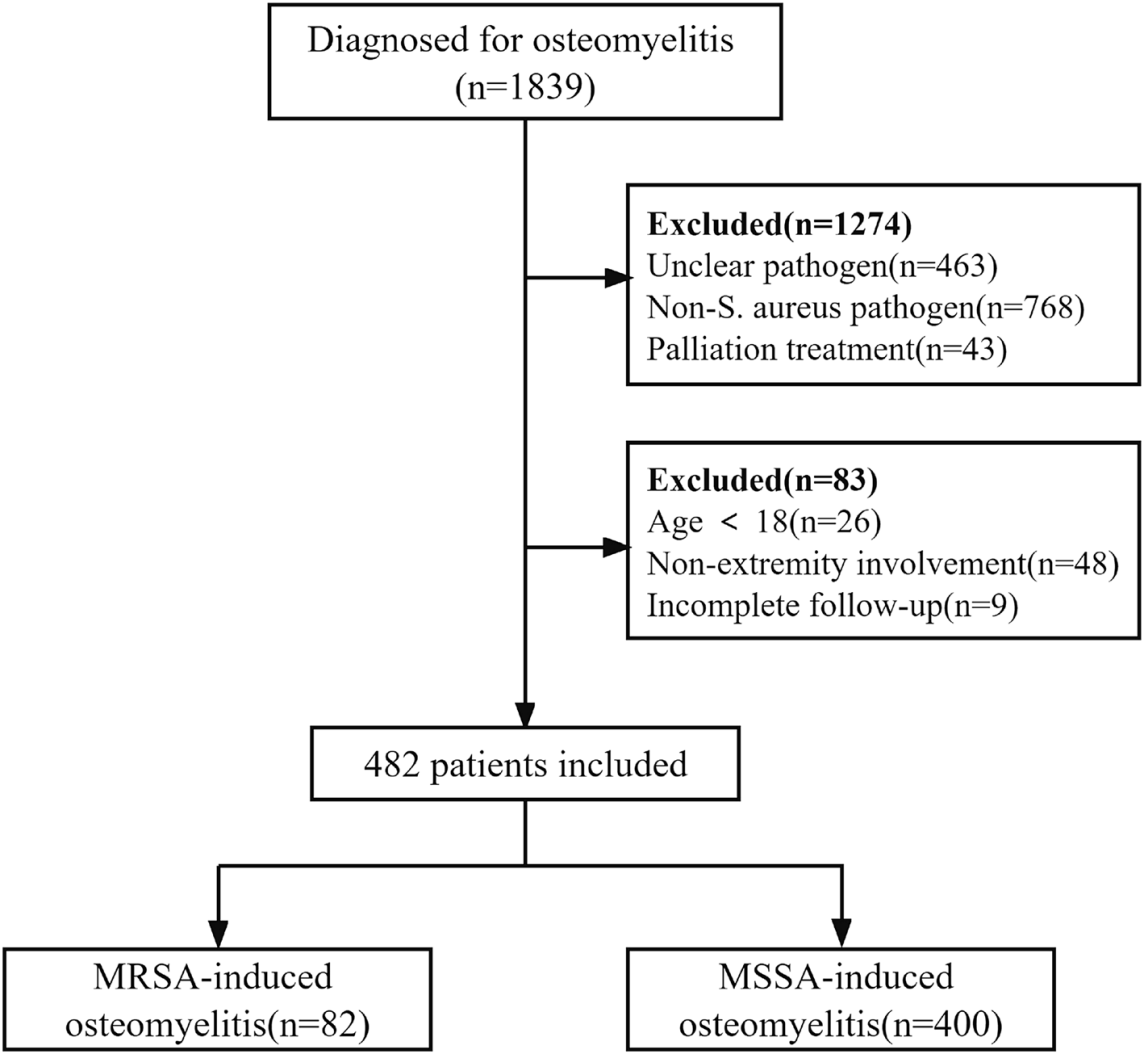
Flowchart of patients included in this study.

**Figure 2:**
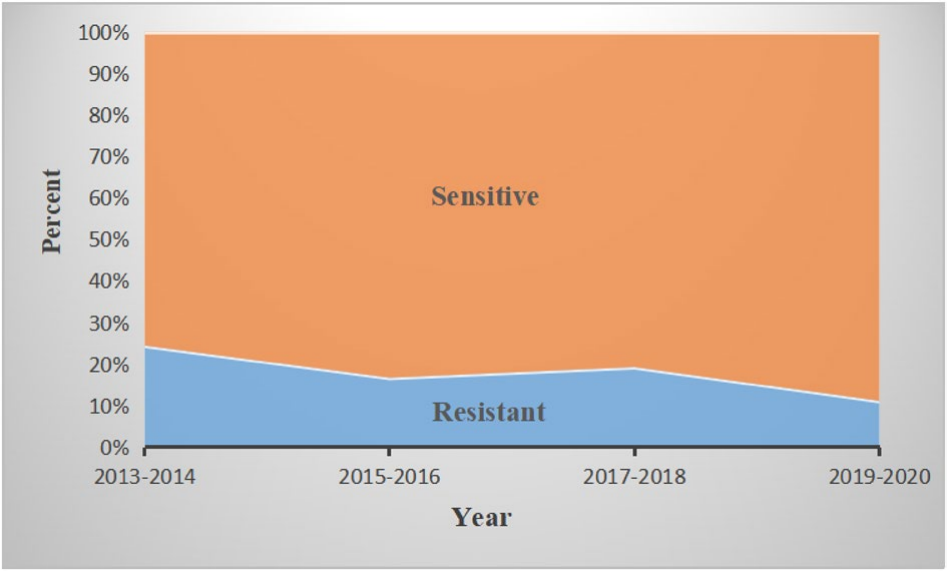
Incidence of methicillin resistance of strains of S aureus cultured from lesions during each year of the study.

From 2013 to 2020, a total of 1839 patients with a discharge diagnosis of osteomyelitis were identified in our initial search during the study period. A total of 1274 osteomyelitis patients were excluded because of infection with an unclear pathogen or a non-*S. aureus* pathogen or received palliation treatment. A total of 74 patients were excluded because they were either below the age of 18 or because of no extremity involvement. Nine patients who had incomplete follow-up data were also excluded. Finally, a total of 482 patients with *S. aureus* extremity osteomyelitis fulfilled our criteria and were included in this study (Fig. 1). The proportion of MRSA and MSSA infection was 17% (82) and 83% (400), respectively. Among which, the proportion of MRSA infection seems to be slightly decreasing in our center recent years (Fig. 2). Patient demographics and clinical characteristics are summarized in Table 1.

**Table 1:** Baseline characteristics of 482 patients with *S. aureus* osteomyelitis.

Variable	Total (N = 482)	Patients with MRSA (N = 82)	Patients with MSSA (N = 400)	P value
Mean age (yrs)	44.35 ± 13.64	41.35 ± 12.84	44.57 ± 13.75	0.051
Age stratification				
18–35 years	132 (27.4)	26 (31.7)	106 (25.5)	0.190
36–60 years	251 (52.1)	45 (54.9)	206 (51.5)	
≥ 61 years	99 (20.5)	11 (13.4)	88 (22.0)	
Gender, male	375 (77.8)	69 (84.1)	306 (76.5)	0.146
Prior debridement (≥ 2)	292 (60.6)	52 (63.4)	240 (60.0)	0.621
Sinus or drains, yes	406 (84.2)	76 (92.7)	330 (82.5)	0.020*
Aetiology				
Traumatic	346 (71.8)	69 (84.1)	277 (69.3)	0.007*
Hematogenous	136 (28.2)	13 (17.9)	123 (30.7)	
Comorbidity				
Tobacco use	207 (42.9)	39 (47.6)	168 (42.0)	0.392
Diabetes	27 (5.6)	4 (4.9)	23 (5.8)	0.804
Systemic diseases	99 (20.5)	24 (29.3)	75 (18.8)	0.036*
CCI score(≥ 3)	117 (24.3)	22 (26.8)	95 (23.6)	0.573
Infection site				
Upper limb	33 (6.8)	7 (8.5)	26 (6.5)	0.773
Femur	128 (26.6)	24 (29.3)	104 (26.0)	
Tibia (Fibula^#^)	279 (57.9)	45 (54.9)	234 (58.5)	
Foot	42 (8.7)	6 (7.3)	36 (9.0)	
Infection duration				
≤ 3 mths	81 (16.8)	10 (12.2)	71 (17.8)	0.046*
3–12 mths	155 (32.2)	36 (43.9)	119 (29.8)	
≥ 12 mths	246 (51.0)	36 (43.9)	210 (52.5)	
C-M host type				
Host A	92 (19.1)	10 (12.2)	82 (20.5)	0.090
Host B	390 (80.9)	72 (87.8)	318 (79.5)	
Polymicrobial, yes	54 (11.2)	12 (14.6)	42 (10.5)	0.335

^#^Patients with fibula osteomyelitis were combined for comparison because of insufficient numbers (n = 5). Significance is denoted by * at the 0.05 level.

Their median age was 44 years with 77.8% (375/482) of males. Of 482 patients, the tibia was most commonly involved (279/482, 57.9%), followed by the femur (128/482, 26.6%), foot (42/482, 8.7%), and upper limb (33/482, 6.8%). The aetiology of trauma accounted for the majority (71.8%) of osteomyelitis patients, with 36.6% cases initially being open fractures and the remaining 28.2% was hematogenous infections. The data showed that patients with osteomyelitis due to MRSA were more likely to have an aetiology of trauma and local sinus or drains, as well as a higher rate of systemic diseases and longer infection duration (Table 1).

### Treatment and infection control results

All patients (100%) received standard debridement and antibiotic treatment. In which, a third generation cephalosporin was empirically used in all patients after debridement. Once the culture results were obtained, patients infected with MRSA were treated with intravenous vancomycin (hospitalization) for 2 weeks and oral linezolid (discharge) for 4 weeks. Patients with MSSA infections were also adjusted based on drug susceptibility, with the same antibiotic duration of intravenous for 2 weeks, then oral for 4 weeks. While additional antibiotic-loaded cement spacer (500 mg gentamicin per 40 mg of PMMA powder mixed with 5 g vancomycin powder) was used for local antibiotic delivery and dead space management in all patients. Moreover, 343 (73%) of patients displayed delayed bone reconstruction after infection control. Patients infected with MRSA underwent more flap coverage surgery and post-operative fixation (Table 2).

**Table 2:** Treatment and outcome of 482 patients with *S. aureus* osteomyelitis.

Variable	Total (N = 482)	Patients with MRSA (N = 82)	Patients with MSSA (N = 400)	P value
				MRSA group vs. MSSA group
Antibiotic treatments, days				
Intravenous	14	14	14	–
Oral	28	28	28	–
Surgery				
Debridement	482 (100)	82 (100)	400 (100)	–
Bone grafts	279 (59.7)	55 (67.1)	224 (56.0)	0.067
Bone transportation	64 (13.3)	10 (12.2)	54 (13.3)	0.859
Flap coverage	79 (16.4)	21 (25.6)	58 (14.5)	0.021
Internal fixation	235 (48.8)	50 (61.0)	185 (46.3)	0.016
Adverse infection control				
Infection persistence (repeated debridement)	66 (13.7)	20 (24.4)	46 (11.5)	0.004^a^
Recurrence	41 (8.5)	5 (6.1)	36 (9.0)	0.516
Total hospital stay, median days	31 (14–112)	37.5 (14–112)	31 (14–82)	< 0.001
Complications	17 (3.5)	7 (8.5)	10 (2.5)	0.015^b^
Pathologic fracture	4 (0.8)	2 (2.4)	2 (0.5)	
Nonunion	5 (1.0)	0 (0)	5 (0.8)	
Amputation	8 (1.7)	5 (6.1)	3 (0.8)	

*MRSA* methicillin-resistant *S*. *aureus*, *MSSA* Methicillin-sensitive *S*. *aureus*.

^a^OR, 2.48 (95% CI 1.38–4.48).

^b^OR, 3.64 (95% CI 1.34–9.86).

Following a 24-month follow up, 13.7% (66/482) of patients failed to respond to initial anti-infection therapy (debridement and antibiotic treatment) and they presented with persistent infection. These patients needed repeated debridement. Of 66 patients with persistent infection, 47 were confirmed by continuous infection signs and symptoms, 19 indicated residual infection before bone defect repair by imaging, pathological and serological tests. Moreover, 8.5% (41/482) had recurrence after treatment completion and a period of infection cure (Table 2).

Univariate analyses indicated osteomyelitis patients with/without methicillin resistance differed significantly in infection persistence (24.4% vs. 11.5%, p = 0.004), no statistical difference was found in recurrence (Table 2). Univariate analyses were used to further determine the association between infection persistence and each potential confounding variable, three variables (Flap coverage, Polymicrobial, Systemic diseases) with a P value < 0.25 were included in the multivariate logistic regression analysis (Table 3). The effect of methicillin resistance remained significant (p = 0.008) following multivariate analysis, the likelihood of developing a persistent infection increased by 2.26-fold (OR: 2.26; 95% CI 1.24–4.13) (Table 4).

**Table 3:** Univariate logistic regression for association of potential confounding factors with infection persistence.

Variable	Infection persistence	
	OR (95% CI)	P value
Age stratification: 18–35 years (ref)		
36–60 years	1.10 (0.60–2.01)	0.77
≥ 61 years	0.79 (0.36–1.76)	0.57
Gender: female versus male (ref)	0.84 (0.44–1.61)	0.60
Aetiology: traumatic versus hematogenous (ref)	0.82 (0.47–1.43)	0.48
Prior debridement: n ≥ 2 versus n ≤ 1	0.81 (0.48–1.36)	0.42
Sinus or drains: yes versus no (ref)	1.22 (0.57–2.57)	0.61
Tobacco use: yes versus no (ref)	1.37 (0.80–2.35)	0.25
Diabetes: yes versus no (ref)	1.10 (0.37–3.30)	0.86
Systemic diseases: yes versus no (ref)	1.70 (0.94–3.05)	0.08*
CCI score: ≥ 3 versus < 3 (ref)	0.699 (0.40–1.24)	0.22
Infection duration: ≤ 3 mths (ref)		
3–12 mths	0.80 (0.37–1.75)	0.90
≥ 12 mths	0.95 (0.47–1.94)	0.71
C-M host Type: host B versus host A (ref)	0.96 (0.50–1.84)	0.89
Polymicrobial: yes versus no (ref)	1.73 (0.84–3.56)	0.13*
Flap coverage: yes versus no (ref)	1.62 (0.86–3.05)	0.14*
Internal fixation: yes versus no (ref)	1.14 (0.68–1.91)	0.63

*OR* odds ratio, 95% CI 95% confidence interval, *CCI* charlson comorbidity index, *ref* reference.

*P < 0.25.

**Table 4:** Analysis of potential effect modifiers for the association of methicillin resistance and infection persistence following multivariate analysis.

Variable	Infection persistence	
	OR (95% CI)	P value
Flap coverage: yes versus no (ref)	1.33 (0.69–2.58)	0.391
Polymicrobial: yes versus no (ref)	1.57 (0.75–3.30)	0.234
Systemic diseases: yes versus no (ref)	1.53 (0.84–2.79)	0.163
Methicillin resistance: MRSA versus MSSA (ref)	2.26 (1.24–4.13)	0.008*

*OR* odds ratio, 95% CI 95% confidence interval, *ref* reference, *MRSA* methicillin-resistant *S*. *aureus*, *MSSA* Methicillin-sensitive *S*. *aureus*.

*P < 0.05.

### Length of hospital stay and complications

Complications were discovered in 3.5% (17/482) of patients (pathologic fracture; 4, nonunion; 5, amputation; 8) at final follow-up. Except for amputees, other patients with complications received surgical intervention again, and all achieved clinical resolution of infections. Of all 482 patients, the total length of hospital stay was 31 (range: 14–112) days. Single factor analyses indicated patients infected with MRSA are more likely to develop a complication (8.5% vs. 2.5%, p = 0.015) and had longer hospital stay (median: 37.5 vs. 31 days, p < 0.001) compared to patients infected with MSSA (Table 2).

## Discussion

In bacterial infectious diseases, MRSA-induced infection is a great concern^17,18^, as well as that in the field of extremity osteomyelitis^1,2,12^. Our data showed the proportion of MRSA was 17% (82/482) in *S. aureus* osteomyelitis, with a slightly downward trend observed in our clinic center. That may attribute to this type of disease received early surgical intervention currently^1,16^, rather than relying on longterm antibiotic treatment as before. While literature reported the proportion of MRSA to be 20.1–30.3%^19–21^. Moreover, the results indicated that MRSA-induced osteomyelitis had poor treatment response after receiving standard surgery and antimicrobial therapy. Previously, the clinical impact of MRSA infections on adult osteomyelitis was unclear^12–14^, our findings may advance the understanding and treatment of extremity osteomyelitis caused by MRSA.

Our study first demonstrated that methicillin resistance has an independent prognosis implication for *S. aureus* osteomyelitis in adult patients. Earlier, the osteomyelitis cohorts reported by West et al*.*^14^ and Gramlich et al*.*^22^ also showed that the failure rate was higher in population of MRSA compare to patients with MSSA. Unfortunately, these reports had relatively small cases and were simple descriptive study. Among which, patients infected with MRSA had more comorbid conditions, and these factors were not adjusted. In this study, we found osteomyelitis patients infected with MRSA were more likely to have a systemic diseases. When these confounding factors were adjusted in the multivariate analysis, the differential outcome were still present between two populations (MRSA/MSSA). The results support the notion that the attributable clinical outcome related to methicillin resistance among patients with *S. aureus* osteomyelitis is real and cannot be solely explained by differences in patient factors. This highlights the challenge implicated in the treatment of extremity osteomyelitis due to MRSA.

Although this cohort received standard debridement and antibiotic treatment, the reasons behind the difference in prognosis is a great concern, and may suggest that a causal pathway for MRSA may be complex. Importantly, both surgical intervention and adjuvant antibiotic therapy are the cornerstone of osteomyelitis treatment success^2,16^. Surgical treatment in particular plays a critical role in improving the local environment, includes the removal of poor vascularized tissue, reducing the bacterial load, and enhancing the antibiotic delivery. Moreover, even for the MRSA pathogen, the regimen of vancomycin and linezolid in this study can establish an effective treatment. Nevertheless, we believe the treatment outcomes more or less reflect factors related to the organism, treatment, and the patient.

The most important and likely cause is the delay in effective antibiotic treatment for MRSA. Obviously, the empirical antibiotic protocol (a third generation cephalosporin) after debridement in this study is typically effective for MSSA and not for MRSA. Further, effective vancomycin therapy was usually adjusted after culture results obtained (3 days are often required after debridement in our hospital). It is generally believed this period (3 days) is crucial for treatments of osteomyelitis, in killing the residual bacteria after debridement; otherwise, the residual plankton bacteria can quickly re-colonize poorly vascularized tissues (forming a biofilm) in this short time frame^23,24^, making it difficult to be cleared. Among bacteremia patients due to MRSA, a highly predictive effect of delayed effective antimicrobial therapy on clinical outcomes had been discovered^25^. Based on these results, it is necessary to detect MRSA early and ensure that appropriate therapy is initiated promptly in patients at risk for MRSA.

The results of this study also raise some concerning questions about the optimal antibacterial strategy for MRSA. However, on this topic to date there is not a conclusion in the literature (including MRSA)^26,27^. Since the work of Waldvogel et al*.*^3^, published in the New England Journal of Medicine in 1970, a treatment duration of at least 4 weeks has been widely advocated. This was based on the comparison outcomes in two groups receiving “intensive” (over 4 weeks) and limited regimen. Since then, antibiotic therapy is usually recommended for 4–6 weeks, so did us in this study. In addition, a Meta-analysis conducted by Li et al*.*^26^ showed no difference in outcomes between intravenous antibiotic and oral antibiotic treatment for osteomyelitis. Currently, oral drugs can penetrate the bone well, including those used in the therapeutic treatment of MRSA (e.g. linezolid)^28–30^. According to our results, more research is needed to determine the optimal antibacterial strategy for osteomyelitis caused by MRSA.

Finally, there is growing concern of vancomycin resistance to *S. aureus*. That may also have a role in the prognosis of patients with MRSA and MSSA infections. Some researchers^31,32^ believe that this issue is underappreciated because the susceptibility breakpoint designated by the CLSI is too high (MIC ≤ 2 mg/L). These reports have described the MRSA strains with either a moderate sensitivity or a high level of resistance to vancomycin^31,33^. Another multicenter, prospective, clinical experiment showed that in patients with MRSA bacteremia treated with vancomycin, clinical success was highly dependent on vancomycin MIC^32^. For MRSA isolates with vancomycin MICs of 0.5 mg/L or less, vancomycin treatment success was 55.6%, while vancomycin MICs of 1–2 mg/L cases were effective in only 9.5% patients. Despite this issue not being found and confirmed in this study, its attributable influence on patients’ prognoses warrants discussion.

In addition to seeking more effective antibiotic therapy for MRSA as described above, the results of this study also have other implications for the treatment of osteomyelitis due to MRSA. First, the results revealed that MRSA-induced osteomyelitis often led to more surgery, prolonged antimicrobial therapy (including length of stay), and potentially more complications. This prognostic data may help to well counsel and prepare patients for treatment. Second, a staged treatment strategy^34^ (delayed bone reconstruction) may be more suitable for patients infected with MRSA, because they often need serial debridement before definitive treatment. Lastly, the delay in effective antimicrobial therapy for MRSA after debridement highlights that radical debridement is critical in treating osteomyelitis patients infected with MRSA; otherwise, the residual bacteria can easily re-colonize on poorly vascularized tissues^23^, failing the treatment.

Our current study also has some limitations. First, the population of MRSA patients was relatively small due to epidemiological reasons. Moreover, not all confounding factors could be considered within the confines of this report. Nevertheless, there are many strengths in this study. To our knowledge, this is the first large cohort used to analyze attributable clinical outcome related to methicillin resistance among adult patients with *S. aureus* osteomyelitis. Meanwhile, osteomyelitis treatment heavily relies on the surgeon’s experience. All patients in this study were treated with standard techniques by the same team of specialists, which eliminated the impacts of subjective variables (e.g. debridement). In the multivariate analysis, adjustments were made for systemic diseases, polymicrobial, local soft tissue condition all known complicating factors for osteomyelitis. Therefore, the results provide information used to counsel and prepare patients for treatment, as well as form the basis of optimize therapy and future research.

## Conclusion

We enrolled a large cohort of *S. aureus* osteomyelitis patients and provided insights into comparison prognosis between populations with/without methicillin resistance. The results of this study highlight the osteomyelitis due to MRSA is a challenge compared to MSSA. The former is more likely to have a poor treatment response, leading to the development of a persistent infection. Patients infected with MRSA often need repeated debridement and prolonged antimicrobial therapy, as well as a longer hospital stay, and more complications. This difference may be explained by delayed effective antibiotic therapy after debridement. Based on the above findings, both treating clinicians and patients should recognize its refractory before treatment, that multiple operations and long-term anti-infection treatment are often needed to cure. Future studies should clarify the optimal antimicrobial strategy in osteomyelitis patients due to MRSA.

## Data Availability

The datasets used or analyse during the current study are available from the corresponding author upon reasonable request.
